# Pediatric TBI: Direct admissions vs. secondary referrals to a hospital: A single‑center, retrospective study

**DOI:** 10.3892/mi.2024.182

**Published:** 2024-07-23

**Authors:** Moosa Allawati, Yahya Al-Kindi, Said Al Jaadi, Tariq Al-saadi

**Affiliations:** 1 College of Medicine and Health Sciences, Sultan Qaboos University, Muscat 123, Sultanate of Oman; 2 Oman Medical Specialty Board, Muscat 130, Sultanate of Oman; 3 Armed Forces Medical Services, Muscat 132, Sultanate of Oman; 4 Department of Neurology and Neurosurgery, Montreal Neurological Institute and Hospital, McGill University, Montreal, QC H3A 2B4, Canada; 5 Department of Neurosurgery, Khoula Hospital, Muscat 127, Sultanate of Oman

**Keywords:** pediatric, traumatic brain injury, interhospital

## Abstract

The present retrospective study was conducted in an aim to examine the differences between pediatric traumatic brain injury (TBI) cases referred to and those admitted directly to the hospital. For this purpose, pediatric patients who presented to a main trauma center with TBI between January, 2015 and December, 2019 were reviewed retrospectively, emphasizing whether they were admitted directly or referred from another center. Data collected included the demographic characteristics of the patients, as well as their presenting complaints and the cause of TBI. A total of 981 cases of pediatric TBI were admitted over the 5-year period. The average age of the patients was 58.1 months for the referred cases and almost 50 months for the patients directly admitted. The male sex accounted for 63.6% of all cases. The most common cause of injury was falling (63.5%). Nausea and vomiting were the most typical presenting symptoms, occurring more among the directly admitted cases (P-value ≤0.05). Mild TBI accounted for 85.3% of the cases, and the most common radiological diagnosis was skull fracture (37.4%) (P-value ≤0.004). The referred patients had a more extended hospital stay (P-value ≤0.001). On the whole, the present study identified 981 cases; the majority of these were direct admissions, and the majority of the severe cases were referred from other healthcare facilities. Further research is required on this topic as only a single hospital was covered herein, and patients were not followed-up after discharge. A multi-center analysis would cover a greater number of patients and would thus provide more substantial data on the topic.

## Introduction

Despite the increase in prevention measures, traumatic brain injury (TBI) continues to be one of the leading causes of global morbidity and mortality among children and is a significant public health concern (1). Furthermore, the implementation of advanced trauma life support and the increase in knowledge about the management of trauma and its sequelae over the past decades were the main reasons for the improvements observed in the outcomes of trauma patients (2).

A local study on childhood traumatic injuries was published in 2018(3). That study demonstrated that out of 795 cases, >50% of the patients were involved in a transport injury, and >11% were involved in falls (3). Annually, ~475,000 children <14 years of age experience a TBI. The majority of these cases (90%) are treated and discharged from the emergency department. Nonetheless, TBIs lead to ~37,000 hospitalizations and 2,685 fatalities each year (4). TBI in a child can have significant consequences, such as delayed or disturbed neurodevelopment (5).

Improvement in trauma care occurred concomitantly with the formation of specialized trauma centers and a trauma system that provided highly specialized care. The main indication for interhospital referrals is that patients require urgent or emergency neurosurgical assessment and intervention (medical/surgical) for acute deadly conditions such as intracranial hemorrhage. For this reason, according to a study that examined interhospital transfers for pediatric neurosurgical cases, TBI with or without intracranial hemorrhage (ICH) was the most common diagnosis (6).

A recent study that examined interhospital referrals for patients with spinal metastasis stated that some hospitals lack resources and request referrals to specialized centers (7). Still, the decision to refer the patient to another hospital must outweigh the risks of delay in proper medical treatment (8,9).

On the other hand, Mowbray *et al* (10) examined the interfacility relocations of those injuries to their emergency department, and 42% of the movements were deemed unnecessary, as those patients did not require specific interventions such as intubation or admission to the intensive care unit. Sathya *et al* mentioned (11) in 2015 that trauma centers could be pediatric trauma centers (PTCs), adult trauma centers (ATCs), or mixed trauma centers (MTCs). They found that injured children who are treated in ATCs or MTCs had a higher in-hospital mortality compared to when they are treated in a PTC (12).

Transferring patients to the nearest hospital can avoidably increase the time to provide definitive care, as the hospital may not be a trauma center, and the impact of this delay in receiving definitive care may significantly affect the outcomes (13). Some studies suggest that transferred patients have a higher mortality rate and a longer period of hospitalization than directly admitted patients (14,15).

Some researchers have tried to determine whether trauma patients initially taken to a non-trauma hospital have a worse outcome than patients who were directly admitted to a trauma center (4). Moreover, previous research has demonstrated significantly lower mortality rates for trauma patients treated in a trauma center compared with those treated in non-traumatic hospitals (16). However, a previous last systematic review could not determine the survival benefit of patients who were directly admitted to a trauma center (12). Patient outcomes are being used widely to measure hospital quality and profile hospital performance (16).

Our neurosurgery and trauma center at Khoula Hospital is a tertiary hospital in the Sultanate of Oman, and it is considered a leading center in neurosurgical services at the national level, with >1,500 patients admitted annually due to traumatic or non-traumatic etiologies (17,18). The present retrospective study aimed to examine and analyze the differences between the referred cases of TBI and those admitted directly to the hospital in terms of the Glasgow Coma Scale (GCS), age at the time of admission and cause of injury. The aim of the present study was to compare these findings with those of other studies published locally or internationally.

## Patients and methods

### Study design

A retrospective study was conducted, in which data on pediatric TBI cases were searched at the neurosurgery and trauma center at Khoula Hospital. Following the acquisition of ethical approval [obtained from the Khoula Hospital's Research Ethics Committee (CODE: PRO01202051)], data were collected using the information system of the hospital. It was deemed by the committee that due to the anonymization of the patients' data and the retrospective nature of the study; individual patient consent was not required.

### Data collection and study subjects

The present study included children examined at the Emergency Department of Khoula Hospital with a diagnosis of TBI between January, 2015 and December, 2019; the patients were of all nationalities. The included pediatric cases were only those with an age <13 years, which is the definition of the national pediatric age group. In addition, the number of patients who came directly to the emergency department and those referred from other hospitals or local health centers were recorded.

The duration of the trauma and the emergency department visit was also recorded, along with the disposition from the emergency department. The mechanisms of head trauma were classified into multiple categories, such as falls, motorcycle accidents, sports-related injuries, etc.

Other collected data included the presenting complaint, GCS upon admission, GCS upon discharge and site of injury. Patients were further classified according to their radiological diagnosis, such as epidural hematoma, subarachnoid hemorrhage, etc. Some of those diagnosed were further classified according to the presence of a single lesion or two lesions in the same patient.

The presence of comorbidities such as diabetes mellitus, hypertension, or pre-existing coagulopathy [indicated by the international normalized ratio (INR)] was also recorded. In addition, the length of hospital stay, the type of treatment received and the complications were recorded for the patients.

### Missing data

Throughout the data collection process, it was observed that certain variables, such as GCS on arrival, presence of coagulopathy and radiological diagnosis, had missing data points. These instances of missing data were carefully documented and reported as a separate entity. The total number of patients in the study was 981; however, specific data points were absent, thus affecting the accurate calculation of percentages and P-values for those variables. The missing data may be attributed to the large sample size, potential errors during data collection, and incomplete records. Although the total number of cases was maintained for overall analysis, each specific cross-table calculation was conducted by excluding the missing data points for the corresponding variables to ensure the precision of P-values and percentages. The total number of missing data points for each variable was computed and reported separately, emphasizing transparency in the management of missing data.

### Statistical analysis

The data were analyzed and processed using the 23rd version of the statistical package for the social sciences (SPSS) software (IBM Corp.). The categorical variables were cross-tabulated using frequency tables and a descriptive bar chart. The Chi-squared and Fisher's exact tests were used to obtain the significance of the association between categorical variables, using a P-value of ≤0.05 as the cut-off for significance. The numerical variables were summarized by their medians, means and ranges, and the categorical variables were described by their counts and relative frequencies. All P-values were two-sided, and a P-value ≤0.05 was considered to indicate a statistically significant difference.

## Results

The demographic characteristics of the patients in the present study, consisting of 981 patients, are presented in Table I. Almost 60% of the patients were admitted directly via the emergency department of the hospital, while almost 40% of the patients were referred from another hospital. Furthermore, the mean age of the referred patients was ~58 months, while the patients who were directly admitted had a younger mean age of almost 50 months. The male patients constituted 63.6% of the study group, while the female patients constituted 36.4% of all the patients (Table I).

The most common mechanism of injury was a fall, which significantly caused head trauma in 623 patients (63.5%), with the majority of the patients being those who came directly to the hospital (69.0%; (P-value ≤0.05); the second most common mechanism was a road traffic accident (RTA), which caused marked head trauma in 180 patients (18.3%), including 106 patients (27.1%) who were referred to the hospital (P-value ≤0.05). The least common cause of injury was head trauma due to bicycle accidents, which occurred in 27 individuals (2.8%); this included 17 patients (4.3%) referred to the hospital (P-value=0.022). Of note, 2.7% of the patients suffered from injuries due to other causes, such as being pushed by someone (Table I).

Furthermore, the most common presenting symptom was nausea/vomiting, which was noted in 598 cases (62.7%), with the majority of the cases being among the patients who came directly to the hospital (70.4%) (P-value <0.05). In addition, the second most common complaint was an altered level of consciousness (29.6%); this occurred in 34.9% of the referred cases and 27.4% of the directly admitted cases with a significant P-value of 0.016 (Table I).

The majority of the patients in the study group were classified as significantly having mild TBI (GCS of 13-15); these consisted of 496 direct cases (91.9%) and 264 referred cases (75.2%). Moreover, out of all the referred cases in the present study, 13.7% of them had a moderate TBI (GCS of 9-12), and 11.1% of them had a severe TBI (GCS <9) (P-value ≤0.05) (Table I).

Of all the referred cases, 50.0% had no coagulopathy (abnormal INR), while 2.8% had an abnormal INR. The directly admitted cases included 38.7% who did not have deranged INR and 2.3% who had abnormal coagulation (P-value=0.005). The coagulopathy (INR) status was unknown in 44.2% of the patients in the study group (Table I).

Detailed information about the radiological diagnoses of the study cases, highlighting significant differences between the referred cases and direct admissions is presented in Table II. Skull bone fracture was the most common diagnosis, observed in 37.4% of the total study group, with a higher incidence in referred cases (43.1%) than in direct admissions (33.7%). Extracranial hematoma, the second most common diagnosis, occurred in 28.7% of all patients, slightly more prevalent in direct admissions (29.0%) than the referred cases (26.8%). Epidural hematoma (EDH) was more common among the referred cases, as 24.1% in one hematoma and 2.4% in one or more hematomas, compared to 10.9 and 0.7% in the directly admitted cases, respectively (P≤0.05). Single subdural hematoma (SDH) was present in 14.5% of the referred cases vs. 6.1% of the direct admissions (P≤0.05). Single subarachnoid hemorrhage (SAH) and single intracerebral hemorrhage (ICH) were also more common in the referred cases (6.6 vs. 2.4% and 14.6 vs. 5.3%, respectively, P≤0.05). Intraventricular hemorrhage was significantly higher in referred cases (2.8%) compared to none in the direct admissions (P≤0.05) (Table II).

On the other hand, surgical management was selected in 4.2% of the directly admitted cases and almost 15% of the referred cases (P-value ≤0.05). Phenytoin was prescribed in the acute setting for 101 patients; 21.4% of the referred cases were prescribed this medication, and a lesser percentage of 3.6% of the directly admitted cases were prescribed this drug (P-value ≤0.05) (Table III). The length of stay in the hospital of both the referred and directly admitted cases is presented in Table IV. It was found that the referred patients had a more extended stay in the hospital in comparison to the directly admitted group; this difference was statistically significant with means of 6 and almost 2 days, respectively. On the other hand, the duration of intensive care unit admission was examined using the same formula, and it found that the difference in the duration of intensive care unit admission between the referred and directly admitted cases was statistically insignificant, with a P-value of 0.366.

**Figure 1:**
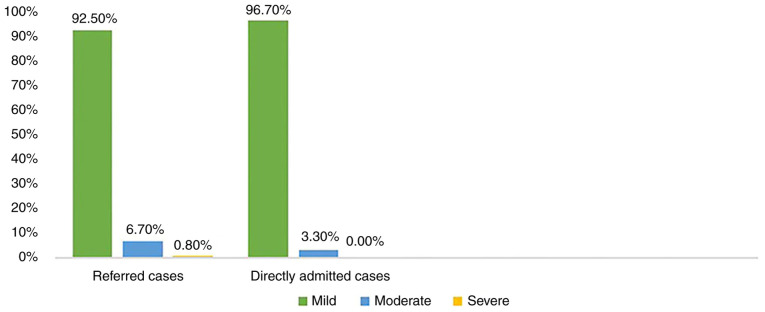
Severity of head trauma among the referred and directly admitted cases upon discharge.

The severity of head trauma the patients had suffered following treatment (i.e., before their discharge from the hospital) is illustrated in Fig. 1. Among the referred cases, 92.5% of the cases were discharged with a GCS of 12-15 (mild), 6.7% had a GCS of 9-12 (moderate), and only 0.8% had a GCS of <9 (severe). On the other hand, among the directly admitted cases, 96.7% were discharged with a GCS of 12-15, 3.3% were discharged with a score that correlates with moderate TBI, and none were discharged with a score of <9.

In the course of the present study, the authors encountered missing data for certain variables. In Table I, there were 40 instances of missing data for GCS on arrival among referred patients and 50 instances among directly admitted patients. Additionally, there were 69 missing cases for the presence of coagulopathy among referred patients and 112 among directly admitted patients. The are also missing data in the data presented in Table II for radiological diagnoses, with specific counts outlined separately. These missing data were included in the overall analysis, but were excluded from specific cross-table calculations to ensure the accuracy of P-values and percentages. The reasons for missing data include the large sample size, potential errors during data collection, and incomplete records.

## Discussion

The main aim of the present study was to compare the differences between the features of referred cases and the cases admitted directly to trauma centers; to the best of our knowledge, this is the first study the Sultanate of Oman with such an aim. Data were extracted from Khoula Hospital's patient hospital information system, one of the country's main trauma centers. The data were gathered by examining the characteristics of each case, including initial GCS and GCS before discharge, as well as age, sex and type of head injury.

The mean ages of the referred patients and the directly admitted cases in the present study were 58.1 and 49.9 months, respectively. The median age of the referred cases was 48 months. Female patients constituted 36.6% of the referred cases and 36.3% of the directly admitted cases. When comparing these demographic statistics to those of another study that examined the characteristics of 400 neurosurgical patients referred to the Pediatric Neurosurgical Service at Texas Children's Hospital, in that study, the median age was 54 months, and 45% of the patients were female (6).

The present study only examined data from one trauma center and focused on pediatric patients with TBI. Time from the site of trauma to the hospital for providing treatment is a crucial factor that affects the management of the patients. Initial assessment (triage) is vital for positive outcomes as it helps deliver the necessary level of care (19). The present study identified 560 patients admitted directly to the trauma center and 390 patients referred from other hospitals and primary healthcare local health centers. In the present study, the majority of 590 direct cases (69.0%) resulted from falls, followed by RTAs at 12.5%. When comparing these findings to those of another survey conducted in the non-pediatric population, it was found that there were 212 severely injured patients referred from outside facilities and 542 severely injured patients that were admitted directly; in that other study, RTAs were the most frequent cause in the cases admitted directly (48.0%), while falls accounted for almost 15% of those cases (19). In that other study, it was concluded that the referred patients were not at an increased risk of mortality, and referred patients were older and had a higher rate of severe TBI compared to the direct cases; it was suggested that this may be due to a reduced rate of recognition of falls and low impact injuries, as a cause of TBI in the elderly (19). In the present study, falls were the primary cause of injury in the directly admitted and referred cases. It is recommended that the recognition of TBI in pediatric and non-pediatric patients, regardless of the causative mechanism of injury, be enhanced to reduce life-long outcomes and enhance the subsequent quality of life (20).

In the present study, the clinical presentation of TBI cases among the two groups was not similar. The most common presentation was nausea and vomiting, which occurred in 70.4% of the direct cases and 50.8% of the referred patients (P-value ≤0.05). For the analysis, GCS was categorized into severe (GCS <9), moderate (GCS 9-12) and mild cases (GCS 13-15). The present study identified that the majority of severe cases (39/46) were referred cases, while the direct cases were 7/46. A total of 11.1% of all referred cases and 1.3% of all direct cases had a severe TBI. Mild TBI among the direct cases constituted 496 out of all 760 mild cases, and 264/760 were referred cases; 91.9% of the direct cases and 75.2% of the referred patients had a mild TBI (P-value ≤0.05). Another study performed in Ohio, USA recorded the GCS twice after trauma, and GCS was categorized as 3, 4-8, 9-15 and unknown (21). They observed that the proportion of referred patients decreased with increasing injury severity scores and escalating head injury severity, as indicated by a lower GCS. The majority of patients with a GCS of 9-15 were referred (83%). Moreover, they identified almost all severe cases were going directly to a level one trauma center, and they had a mortality of 61.3% (21). In contrast to the findings in the present study, severe GCS was the category that was the least likely to be admitted directly to the center (P-value ≤0.05).

As regards the types and radiological diagnoses of TBI in the present study, the present study included the occurrence of both intracranial and extracranial hematomas after trauma. The most common radiological diagnoses among the whole study group were skull bone fracture, single extracranial hematoma and single EDH, which were observed in a total of 363 (37.4%), 273 (28.1%) and 156 (16.1%) cases, respectively. Among the referred patients, skull bone fracture occurred in 43.1% of the cases, and extracranial hematoma occurred in 26.8% of the cases. By contrast, 2 SDH, SAH and ICH lesions only occurred in 0.3% of the cases. Another study examined the causes for the conveyance of neurosurgical emergency cases to five academic neurosurgery departments in Cook County, IL, USA (21). Over a period of 2 months, 230 emergent referrals occurred. The most prevalent diagnoses that required referral were parenchymal ICH (75 patients, 33%), SAH (66 patients 28%) and SDH/EDH (35 cases, (15%) (21).

In addition, the present study demonstrated that the presence of a coagulation defect was not stated in the patients' files in 434 cases. However, out of all the referred cases, half (50.0%) did not have a coagulopathy proven by the INR, while 9 referred cases (2.8%) did have it. On the other hand, out of all directly admitted cases, 38.7% did not have a proven coagulopathy, while only 2.3% had a coagulopathy.

As regards the management of pediatric TBI cases in the present study, the majority underwent conservative treatment. Of note, 85.1% of the referred trauma cases and 95.8% of the directly admitted cases were treated in this manner, while only 14.9% of the referred cases and 4.2% of the directly admitted cases required neurosurgical intervention. Another previous study examined the conveyance requests for pediatric patients diagnosed with various neurosurgical diseases at Texas Children's Hospital; they found that out of 400 conveyance admissions, 77 patients (19.3%) required neurosurgical intervention, with the majority requiring a cranial procedure (66.2%) (6). Another study that investigated the referral of neurosurgical patients of all ages found that out of 984 patients who arrived at their hospital, 42.5% underwent neurosurgical operative or endovascular intervention (22). Additionally, a study published in 2014 compared 212 referred and 542 directly admitted blunt multiply injured patients and found that directly admitted patients were more frequently operated on within 12 h after admission (40.4%) than referred patients (27.8%), concluding that interhospital referral does not impact the outcome of blunt trauma patients (19). In the present study, phenytoin was administered in 21.4% of the referred cases and 3.6% of the directly admitted cases.

In the present study, the referred cases spent a longer time in the hospital than the directly admitted cases (6.03 vs. 1.88) days (P-value <0.001). This is in contrast to the findings of another study, which demonstrated no difference in the duration of hospital stay (11.3 vs. 13.8 days) (19); however, the present study did not find any significant differences in the duration of intensive care unit admission.

The data presented herein indicate that, among the 981 patients, falls were the most prevalent cause of injury, occurring more frequently among the directly admitted patients. Nausea/vomiting was common in both groups, with the majority experiencing mild injuries (GCS of 13-15). The most frequent radiological finding was a skull fracture, observed in >350 patients (37.4%), with a higher incidence among direct cases (33.7%). The comparison of referred and directly admitted cases is essential for investigating the causes of referrals to trauma centers, evaluating the features and management after the referral, and comparing it with the directly admitted cases.

The present study has several limitations which should be mentioned. Data were gathered from a prospectively collected database and were restricted to a single center. Detailed clinical information and patient status outside the facility and during decision-making were limited. Moreover, more detailed information is required about any intervention or diagnostic methods performed before the patients were referred. The need for a control group and the number of patients who succumbed before they were referred can also limit the analysis. Most of the patients were referred to a nearby hospital for follow-up after being discharged from Khoula Hospital, which limited the possibility of long-term follow-up in the present study. Further multi-center analyses and cohort studies are thus required for a good comparison of the outcomes. The lack of long-term data for the patients in the present study can influence the outcome of patients who may have deteriorated after their discharge from the hospital, and such data need to be obtained in order to avoid bias in the outcome.

## Figures and Tables

**Table I:** Demographic characteristics of the study group.

Parameter	Referred cases, n=391	Directly admitted, n=590	Total, n=981	P-value
Age, months				
Frequency, n (%)	391/981 (39.9%)	590 (60.1%)	981 (100.0%)	-
Mean ± standard deviation	58.1±40.6 months	49.9±38.2 months	-	
Sex, n (%)				
Male	248 (63.4%)	376 (63.7%)	624 (63.6%)	0.977
Female	143 (36.6%)	214 (36.3%)	357 (36.4%)	
Cause of injury, n (%)				
Fall	216 (55.2%)	407 (69.0%)	623 (63.5%)	≤0.05
Road traffic accident	106 (27.1%)	74 (12.5%)	180 (18.3%)	≤0.05
Hit by heavy objects	28 (7.2%)	40 (6.8%)	68 (6.9%)	0.910
Missing data^a^	1	0	1	
Bicycle accident	17 (4.3%)	10 (1.7%)	27 (2.8%)	0.022
Sport-related injuries	13 (3.3%)	31 (5.3%)	44 (4.5%)	0.203
Others^b^	6 (1.5%)	20 (3.4%)	26 (2.7%)	0.117
Presenting complaints, n (%)				
Altered consciousness	132 (34.9%)	158 (27.4%)	290 (30.4%)	0.016
Missing data^a^	13	13	26	
Nausea/vomiting	191 (48.8%)	407 (68.9%)	598 (60.9%)	≤0.05
Missing data	15	12	27	
Headache	30 (7.6%)	75 (12.7%)	105 (10.7%)	0.021
Missing data^a^	15	12	27	
Visible facial/head injury	82 (20.9%)	92 (15.6%)	174 (17.7%)	0.027
Missing data^a^	15	12	27	
GCS (on arrival), n (%)				
Mild (13-15)	264 (75.5%)	496 (84.1%)	760 (77.5%)	≤0.05
Moderate (9-12)	48 (13.7%)	37 (6.9%)	85 (9.5%)	
Severe (<9)	39 (11.1%)	7 (1.3%)	46 (5.2%)	
Missing data^a^	40	77	117	
Presence of coagulopathy, n (%)				
Unknown	152 (47.2%)	282 (59.0%)	434 (54.3%)	0.005
Absent	161 (50.0%)	185 (38.7%)	346 (43.3%)	
Present	9 (2.8%)	11 (2.3%)	20 (2.5%)	
Missing data^a^	69	112	181	

^a^Percentages for each category were calculated based on cases with available data, excluding those with missing information;

^b^others includes injuries caused by such as being pushed by someone. GCS, Glasgow Coma Scale.

**Table II:** Radiological diagnosis of the cases.

Radiological diagnosis	No. of hematomas	Referred cases, n=391; n (%)	Directly admitted, n=590; n (%)	Total n=981; n (%)
EDH^a^	None	280 (73.5%)	518 (88.4%)	798 (82.5%)
(P-value ≤0.05)	1	92 (24.1%)	64 (10.9%)	156 (16.1%)
	2 or more	9 (2.4%)	4 (0.7%)	13 (1.3%)
	Missing data^c^	10	4	14
SDH^a^	None	324 (85.3%)	550 (93.7%)	874 (90.4%)
(P-value ≤0.05)	1	55 (14.5%)	36 (6.1%)	91 (9.4%)
	2 or more	1 (0.3%)	1 (0.2%)	2 (0.2%)
	Missing data^c^	11	3	14
SAH^a^	None	353 (93.1%)	572 (97.4%)	925 (95.8%)
(P-value ≤0.05)	1	25 (6.6%)	14 (2.4%)	39 (4.0%)
	2 or more	1 (0.3%)	1 (0.2%)	2 (0.2%)
	Missing data^c^	12	3	15
ICH/contusion^a^	None	326 (85.1%)	554 (94.5%)	880 (90.8%)
(P-value ≤0.05)	1	56 (14.6%)	31 (5.3%)	87 (9.0%)
	2 or more	1 (0.3%)	1 (0.2%)	2 (0.2%)
	Missing data^c^	8	4	12
Cerebellar hemorrhage^a^	None	381 (99.2%)	581 (99%)	962 (99.1%)
	-	3 (0.8%)	6 (1.0%)	9 (0.9%)
	Missing data^c^	7	3	10
IVH	None	310 (97.2%)	479 (100%)	789 (98.8%)
(P-value ≤0.05)	-	9 (2.8%)	0 (0.0%)	9 (1.2%)(%)
	Missing data^c^	72	111	183
Extracranial hematoma^a^	None	276 (71.9%)	416 (70.9%)	692 (71.2%)
(P-value ≤0.05)	1	103 (26.8%)	170 (29.0%)	273 (28.1%)
	2 or more	5 (1.3%)	1 (0.2%)	6 (0.7%)
	Missing data^c^	7	3	10
Skull bone fracture^b^	None	218 (71.9%)	389 (70.9%)	692 (91.2%)
	-	165 (43.1%)	198 (33.7%)	363 (8.8%)
	Missing data^c^	8	3	13

Data were analyzed using

^a^Fisher's exact test and

^b^the Chi-squared. A P-value=<0.05 was considered to indicate a statistically significant difference. No reported P-value indicates a statistically insignificant result.

^c^Percentages for each category were calculated based on cases with available data, excluding those with missing information. EDH, epidural hematoma; SDH, subdural hematoma; ICH, intracranial hemorrhage; IVH, Intraventricular hemorrhage.

**Table III:** Management of head trauma in the study group.

Parameter	Referred cases, n=316; n (%)	Directly admitted, n=479; n (%)	Total, n=795; n (%)	P-value
Type of treatment				≤0.05
Observation	269 (85.1)	459 (95.8)	728 (91.6)	
Surgery	47 (14.9)	20 (4.2)	67 (8.4)	
Phenytoin administration	n=374 80 (21.4)	n=582 21 (3.6)	n=956 101 (10.6)	≤0.05

**Table IV:** Length of hospital stay and/or ICU admission among the two study groups.

Parameter	Referred case (R) or direct access to the hospital (D)	Mean (no. of days)	P-value^a^
Length of hospital stay	Referral	6 days	<0.001
	Direct admission	2 days	
Duration of ICU admission	Referral	6-7 days	0.366
	Direct admission	4-5 days	

^a^Data were analyzed using the Mann-Whitney test for non-parametric data. ICU, intensive care unit.

## Data Availability

The datasets used and/or analyzed during the current study are available from the corresponding author on reasonable request.
